# Depression and Inflammation: Is There any Role for Biomarkers?

**Published:** 2019

**Authors:** Shahin Akhondzadeh

**Affiliations:** Psychiatric Research Center, Roozbeh Hospital, Tehran University of Medical Sciences, Tehran, Iran

Despite the advent of several antidepressant medications, treatment of Major Depressive Disorder (MDD) is still far from optimal 1–3. A large proportion of patients with MDD do not respond to their first medication. To achieve favorable response, these patients are generally treated with either switching to another treatment or augmentation therapy. In the recent decade, several augmentative strategies for treatment of MDD have been developed. Some of these treatment modalities focus on recently developed hypotheses of pathophysiological processes in patients with MDD 1–3. These mainly include immune system dysfunction, hypothalamic-pituitary-adrenal (HPA) axis and metabolic derangements, impaired neuroprotection, or neuroinflammation 1–3.

Growing body of evidence suggests that inflammation is implicated in the pathophysiology of MDD 4–6. Sickness behavior which is a result of inflammatory activation, shares many clinical features such as anhedonia, anorexia, irritability, and mild cognitive problems with MDD^4^–^6^. Several studies have shown an elevation of proinflammatory cytokines [particularly IL-6 and Tumor Necrosis Factor (TNF-α)] in patients with MDD^7^. A large body of research now suggests that depression is associated with a low-grade, chronic inflammatory response and is accompanied by increased oxidative stress.

- depression frequently is comorbid with many inflammatory illnesses
- increased inflammatory biomarkers are associated with major depressive disorder (MDD)
- exposure to immunomodulating agents may increase the risk of developing depression
- stress can activate proinflammatory pathways
- antidepressants can decrease inflammatory response
- inhibition of inflammatory pathways can improve mood 4–7.

IL-6 is one of the most widely studied cytokines in patients with MDD^8^,^9^. In addition to elevation of this cytokine in patients with MDD, relation of IL-6 concentration to severity of depression, a shift in circadian rhythm^8^,^9^, and a reduction in its concentration in response to antidepressants have been shown in several studies.

Previous studies have already shown that elevated levels of inflammation are associated with poor response to antidepressants. The scientists found that they could pinpoint a threshold and precisely predict which patients would respond to conventional antidepressants. None of the patients with MIF and IL-1β levels above the threshold responded to the antidepressants most often prescribed. Those with inflammation levels below the threshold would likely respond. One reason for the lack of predictive biomarkers in MDD is that little is known with absolute certainty about how antidepressants improve mood. All currently approved medications for depression act in a similar way, increasing the availability of monoamine neurotransmitters like serotonin in the brain. Psychiatrists continue to search for biomarkers to help guide therapy and, potentially, improve chances of discovering new drugs 9.

In conclusion, the link between depression and the body's inflammatory response continues getting stronger, with more research showing an ever-tighter correlation.
